# Explainable AI-Based Skin Cancer Detection Using CNN, Particle Swarm Optimization and Machine Learning

**DOI:** 10.3390/jimaging10120332

**Published:** 2024-12-22

**Authors:** Syed Adil Hussain Shah, Syed Taimoor Hussain Shah, Roa’a Khaled, Andrea Buccoliero, Syed Baqir Hussain Shah, Angelo Di Terlizzi, Giacomo Di Benedetto, Marco Agostino Deriu

**Affiliations:** 1 Department of Research and Development (R&D), GPI SpA, 38123 Trento, Italy; syedadilhussain.shah@gpi.it (S.A.H.S.); andrea.buccoliero@gpi.it (A.B.); angelo.diterlizzi@gpi.it (A.D.T.); 2 PolitoBIOMed Lab, Department of Mechanical and Aerospace Engineering, Politecnico di Torino, 10129 Turin, Italy; 3 Department of Computer Engineering, University of Cádiz, 11519 Puerto Real, Spain; roaa.khaled@gm.uca.es; 4 Human Science Department, Università degli studi di Verona, Lungadige Porta Vittoria, 17, 37129 Verona, Italy; 5 Department of Computer Science, COMSATS University Islamabad (CUI), Wah Campus, Wah 47000, Pakistan; bakirhussain6@gmail.com; 6 7HC SRL, 00198 Rome, Italy; giacomo@7hc.tech

**Keywords:** transfer learning, feature extraction, feature selection, particle swarm optimization, ablation, subspace KNN, medium Gaussian SVM, explainable-AI

## Abstract

Skin cancer is among the most prevalent cancers globally, emphasizing the need for early detection and accurate diagnosis to improve outcomes. Traditional diagnostic methods, based on visual examination, are subjective, time-intensive, and require specialized expertise. Current artificial intelligence (AI) approaches for skin cancer detection face challenges such as computational inefficiency, lack of interpretability, and reliance on standalone CNN architectures. To address these limitations, this study proposes a comprehensive pipeline combining transfer learning, feature selection, and machine-learning algorithms to improve detection accuracy. Multiple pretrained CNN models were evaluated, with Xception emerging as the optimal choice for its balance of computational efficiency and performance. An ablation study further validated the effectiveness of freezing task-specific layers within the Xception architecture. Feature dimensionality was optimized using Particle Swarm Optimization, reducing dimensions from 1024 to 508, significantly enhancing computational efficiency. Machine-learning classifiers, including Subspace KNN and Medium Gaussian SVM, further improved classification accuracy. Evaluated on the ISIC 2018 and HAM10000 datasets, the proposed pipeline achieved impressive accuracies of 98.5% and 86.1%, respectively. Moreover, Explainable-AI (XAI) techniques, such as Grad-CAM, LIME, and Occlusion Sensitivity, enhanced interpretability. This approach provides a robust, efficient, and interpretable solution for automated skin cancer diagnosis in clinical applications.

## 1. Introduction

Skin cancer is usually caused by UV radiation from sunlight or tanning beds, which leads to the unconstrained enlargement of unusual skin cells [1]. It is challenging to accurately provide an exact number of skin cancerous cases, but the International Agency for Research on Cancer (IARC) has given an estimation of around 18 million new cancer cases and approximately 9 million cancer deaths around the world in 2018, including skin cancer patients [2]. In addition, the four most common primary kinds of skin cells are Basal cell carcinoma (BCC), squamous cell carcinoma (SCC), melanoma (MEL), and Merkel cell carcinoma (MCC), which usually lead to skin cancer [3].

Skin lesions can develop during infancy and persist into adulthood [4,5,6,7,8]. These lesions often arise from infections and inflammation at an early age [4,9,10]. While some rashes are benign, others can lead to malignant lesions, potentially causing severe issues like neural tube defects [11,12]. Therefore, it is crucial to provide proper care for neonates in Neonatal Intensive Care Units (NICUs) to prevent skin diseases from progressing to dangerous malignant states that could impact the growth and development of the neonatal brain and body [13].

The early and precise diagnosis of skin cancer is very crucial for early and effective treatment to result in improved patient outcomes. Traditional methods of diagnosis heavily rely on visual examination by dermatologists. Considering this, these kinds of methods of diagnosis are time-consuming, sometimes subjective, and often require specialty for right diagnosis [14,15,16]. Consequently, there is a need for an artificial intelligence (AI) tool to detect early diagnosis and assist the dermatologists in their decisions [17,18,19].

For several reasons, information technology (IT) is used to identify skin cancer. It offers sophisticated image processing methods that help with automatic lesion detection and categorization [20,21]. The use of various features by AI-based cancer detection algorithms can help in precise diagnosis. These characteristics can include shape features, like the irregularity of tumors, texture features, which capture variations in tissue patterns, intensity features, which reflect the statistical properties of pixel intensities, local features, which concentrate on regions of interest, and contextual features, which consider spatial relationships between various structures [22,23]. Although, conventional machine-learning approaches in the domain of skin cancer diagnosis typically employ extracting features from skin-disease images for the classification process [24]. For example, the seven-Point Checklist, the ABCD Rule, and the as-well-as Menzies Method are the most conventionally used methods for extracting various features from skin disease images [25,26].

Recent advancements in AI and machine learning (ML) have shown promising results in medical image analysis. In particular, deep learning (DL) has emerged at the forefront of these advancements, demonstrating exceptional capabilities in object detection tasks [27,28,29]. Within the domain of DL, researchers have introduced numerous convolutional neural network (CNN) architectures, such as Xception [30], VGG [31], and GoogleNet [32]. These architectures offer various capabilities tailored to specific problems and techniques like transfer learning, which have simplified the process for both experts and non-experts. Consequently, pretrained CNN models are increasingly being used for tasks such as skin lesion classification, requiring fewer samples to achieve effective results [33,34,35,36,37].

To address these challenges, this study aims to develop a comprehensive, interpretable, and efficient AI pipeline for automated skin cancer diagnosis. Specifically, this research focuses on combining transfer learning, feature optimization, and explainable AI (XAI) techniques to enhance diagnostic performance and transparency.

The novelty of our proposed pipeline lies in its application-focused integration of a single CNN model, Xception, with advanced feature optimization and explainable AI techniques. This study demonstrates how combining the Xception model with an optimizer such as PSO addresses clinical challenges like computational inefficiency, limited interpretability, and inconsistent accuracy, achieving state-of-the-art classification performance. Here, performance refers to a combination of high classification accuracy, computational efficiency, and clinical interpretability, all critical for real-world applications. The inclusion of explainable AI techniques, including Grad-CAM, LIME, and Occlusion Sensitivity, ensures the model’s predictions are interpretable, enhancing trust and usability in clinical settings.

The major contributions of this research are as follows:

1. Application-Oriented AI Pipeline: Developed a complete AI pipeline for skin cancer detection, tailored to clinical needs by integrating preprocessing, Xception-based transfer learning, feature extraction, feature selection, and conventional ML models.
2. Optimization of Xception for Clinical Use: Enhanced the Xception model’s performance by balancing computational efficiency and accuracy, ensuring feasibility in resource-constrained environments.
3. Feature Space Optimization for Practicality: Applied Particle Swarm Optimization (PSO) [38] to reduce feature dimensions from 1024 to 508, improving computational efficiency without sacrificing critical diagnostic information.
4. Clarified Performance Outcomes: Achieved state-of-the-art classification accuracy (98.5% and 86.1% on ISIC 2018 and HAM10000 datasets, respectively), reduced computational overhead through dimensionality reduction, and ensured model interpretability with Grad-CAM, LIME, and Occlusion Sensitivity, thereby demonstrating suitability for clinical decision support systems.

In the upcoming sections, Section 2 of this paper elaborates the related works while Section 3 discusses experimental datasets and the proposed methodology, focusing on modifications to the pretrained CNN-based Xception network for transfer learning, robust feature extraction and selection using the PSO algorithm, and various machine-learning classifiers. Section 4 and Section 5 present the results and discussion. Finally, the explainable AI-based results and conclusion are provided in Section 6 and Section 7.

## 2. Related Works

Skin cancer detection has become a focal point of research, leveraging advancements in artificial intelligence (AI) and machine learning (ML) to address the limitations of traditional diagnostic methods. AI-driven techniques aim to enhance diagnostic accuracy, reduce reliance on subjective assessments, and improve clinical decision-making. This section reviews key contributions in the field, focusing on their methodologies, datasets, and outcomes, while identifying challenges that persist.

### 2.1. Transfer Learning and Pretrained Models

Transfer learning has proven effective in addressing the challenges of limited data availability in skin cancer detection, as highlighted by various studies summarized in Table 1. For instance, Al-Rasheed, et al. [39] introduced a novel approach combining conditional generative adversarial networks (CGANs) for generating realistic dermoscopic images with an ensemble of finely tuned transfer learning models. By training these models on both balanced and unbalanced datasets, they addressed the challenges posed by dataset imbalance in skin lesion classification. Individually, their models achieved accuracies of 92% for VGG16 and ResNet50, and 92.25% for ResNet101 when augmented data were included. When these models were used collectively in an ensemble configuration, the accuracy improved further to 93.5%, highlighting the benefits of combining multiple models for enhanced performance. This strategy demonstrated superior results in skin lesion classification compared with earlier methods, emphasizing the potential of combining advanced data augmentation and ensemble learning to improve diagnostic accuracy. However, the approach lacked interpretability tools to explain model predictions, a critical aspect for clinical applications, which is addressed in our proposed methodology.

Raju, et al. [40] proposed a fine-tuned deep neural networks pipeline for skin cancer classification, utilizing the HAM10000 dataset to evaluate performance. Their methodology employed two prominent pretrained models, InceptionV3 and DenseNet201, fine-tuning them to adapt to the binary classification task of identifying benign and malignant lesions. The InceptionV3 model achieved a testing accuracy of 86.82%, while DenseNet201 slightly outperformed it with an accuracy of 87.72%. The study emphasized the importance of transfer learning for feature extraction, particularly when dealing with imbalanced datasets. However, while the results were promising, the models did not incorporate optimization techniques for feature selection or interpretability tools to enhance clinical applicability. This leaves room for further improvement in balancing performance with computational efficiency and explainability, as demonstrated in our proposed methodology.

Similarly, Ali, et al. [41] conducted an extensive comparison of renowned transfer learning CNN models with a custom-designed deep convolutional neural network (DCNN) for skin cancer classification using the HAM10000 dataset. Their study involved robust data augmentation techniques, including rotation, flipping, and scaling, to address dataset imbalances and improve the training process. Among the models tested, their custom-designed DCNN achieved the highest accuracy of 91.43%, surpassing popular CNN architectures such as AlexNet, ResNet, and VGG-16. The custom DCNN demonstrated enhanced performance through tailored architectural adjustments, optimizing it for the specific task of lesion classification. While the study highlighted the benefits of customization and augmentation, it lacked advanced feature selection methods and explainable AI techniques, which are critical for understanding model predictions in clinical settings. These limitations underscore the significance of incorporating such tools, as demonstrated in our proposed methodology, to build more interpretable and efficient diagnostic systems.

Akilandasowmya, et al. [42] introduced the SCSO-ResNet50-EHS-CNN pipeline for skin cancer diagnosis, combining Sand Cat Swarm Optimization (SCSO) and ResNet50 for feature extraction with Enhanced Harmony Search (EHS) for feature optimization. Evaluated on the ISIC 2019 dataset, the method achieved 92% accuracy, 93.9% sensitivity, and 85.5% specificity. While the approach effectively optimized features using ensemble classifiers like SVM and k-NN, it faced challenges in achieving balanced specificity and lacked explainable AI tools for model interpretation. Compared with our proposed methodology, this work demonstrates the potential of optimization techniques but falls short in its interpretability and generalization, highlighting the need for integrating explainability to enhance clinical relevance.

In addition to CNN models, vision transformers (ViT) have emerged as a promising approach in medical imaging due to their ability to model long-range dependencies within images. Unlike traditional CNNs, which rely on localized receptive fields to extract features, ViTs employ self-attention mechanisms to capture global relationships between image patches. This capability makes them particularly effective in tasks requiring fine-grained feature representation, such as skin lesion classification. However, it is important to note that both CNNs and ViTs can be computationally expensive, especially when dealing with high-resolution medical images and large datasets. ViTs, in particular, often require extensive pre-training on large datasets and significant computational resources during fine-tuning, which may pose challenges for deployment in resource-constrained environments. Similarly, hybrid ViT-CNN models increase computational overhead further, as they combine the complexities of both architectures [43,44,45,46,47].

In another study, Ahmad, et al. [48] utilized the HAM10000 dataset to classify benign and malignant skin lesions, achieving over 90% accuracy on certain lesion types using ViT and EfficientNet. However, challenges in generalization and class imbalance were noted, emphasizing the need for fine-tuning ViT models for high-resolution medical images. The research underscores the value of hybrid ViT-CNN approaches for improving diagnostic performance and efficiency.

Further, Saha, et al. [49] integrated ViT with CNNs, including MobileNet and Xception, for skin lesion classification using the ISIC 2019 dataset. By combining segmentation techniques with hybrid models, the study achieved an accuracy of 91.2%, demonstrating robust performance in distinguishing benign and malignant lesions. While effective, limitations include computational overhead and potential challenges in scalability to larger datasets, highlighting areas for optimization in future research.

**Table 1:** Comparison of recent studies on skin cancer classification, highlighting their focus, key contributions, datasets used, methods applied, and achieved accuracies.

Study	Focus	Key Contributions	Dataset	Methods Used	Accuracy (%)
Ali, Miah, Haque, Rahman and Islam [41]	Enhanced deep CNN with transfer learning for skin cancer classification	Custom CNN architecture with data augmentation achieves superior performance	HAM10000	Transfer Learning, Data Augmentation	93
Raju, Hemalatha, Goli, Yuvananda, Karthik and Krishna [40]	Transfer learning with DenseNet201 and InceptionV3 models	Fine-tuned transfer learning with notable accuracy improvements	HAM10000	Transfer Learning, Fine-tuning	86.82–87.72
Al-Rasheed, Ksibi, Ayadi, Alzahrani, Zakariah and Ali Hakami [39]	Ensemble transfer learning models with CGAN augmentation	High classification accuracy using GAN-based augmentation	ISIC 2019	Ensemble Learning, CGAN	92–93.5
Akilandasowmya, Nirmaladevi, Suganthi and Aishwariya [42]	Dimensionality reduction with SCSO and ResNet50	Improved accuracy with dimensionality reduction and ensemble classifiers	ISIC 2019, Kaggle Skin Cancer	ResNet50, SCSO, Ensemble Classifiers	92.035–94.238
Ahmad, Alsulami and Alqurashi [48]	Skin lesion classification using ViT and CNNs	Demonstrated high accuracy with transfer learning and ViT; analyzed performance on HAM10000	HAM10000	Vision Transformers, EfficientNet, MobileNet	~90%
Saha, Joy and Majumder [49]	Hybrid approaches for segmentation and classification	Combined ViT with CNNs for improved segmentation and diagnostic accuracy	ISIC 2019	Vision Transformers, MobileNet	91.2%

### 2.2. Explainable AI in Dermatology

The lack of interpretability in deep-learning models has driven research towards explainable AI methods such as Grad-CAM and LIME. Selvaraju, et al. [50] and Ribeiro, et al. [51] showed that these techniques enhance trust in AI models by providing visual explanations of their predictions, a crucial feature for medical applications.

### 2.3. Challenges Identified

Despite significant advancements in AI-based skin cancer detection, several challenges persist. One major issue is the limited availability of diverse datasets, which often leads to overfitting and hinders the generalization of models to new cases. Class imbalance further exacerbates this problem, as the underrepresentation of certain lesion types can result in biased predictions. Additionally, hyperparameter optimization remains a computationally intensive and time-consuming process, making it challenging to fine-tune models effectively. The inherent complexity of deep-learning models, with their multi-layered architectures, complicates interpretability, making it difficult to explain classification outcomes and build trust with clinicians. Moreover, many models struggle with generalization, performing well on training data but failing to maintain accuracy on unseen datasets, particularly in medical imaging. The lack of explainability in AI models further limits their clinical adoption, as their “black-box” nature raises concerns among healthcare practitioners. Finally, ensuring the seamless integration of AI systems into clinical workflows remains a significant hurdle, requiring compatibility with existing diagnostic processes and tools. Addressing these challenges is essential to fully harness the potential of AI in skin cancer detection and enhance its utility in real-world clinical settings.

## 3. Materials and Methods

The main objective of this study was to develop a robust approach for classifying skin cancer into benign and malignant categories. This research emphasizes the importance of transfer learning, feature extraction, and selection techniques to enhance classification accuracy. Three experimental studies were conducted to achieve this goal, each addressing different aspects of skin disease classification:

- Experiment 1: The architecture of a pretrained Xception-Net was modified by adding global average pooling and dense layers with varying neuron configurations. This enhanced architecture was used for direct classification of benign and malignant lesions using transfer learning.
- Experiment 2: Features were extracted from the trained Xception-Net, and these feature sets were evaluated using multiple machine-learning classifiers, including SVM, KNN, and ensemble models. This experiment provided insights into the effectiveness of integrating deep feature extraction with conventional classifiers.
- Experiment 3: Robust feature sets extracted from the Xception-Net were further refined using Particle Swarm Optimization (PSO) [38] to reduce dimensionality. The optimal feature sets were then classified using Subspace KNN, demonstrating significant improvements in accuracy, sensitivity, and specificity.

**Figure 1:**
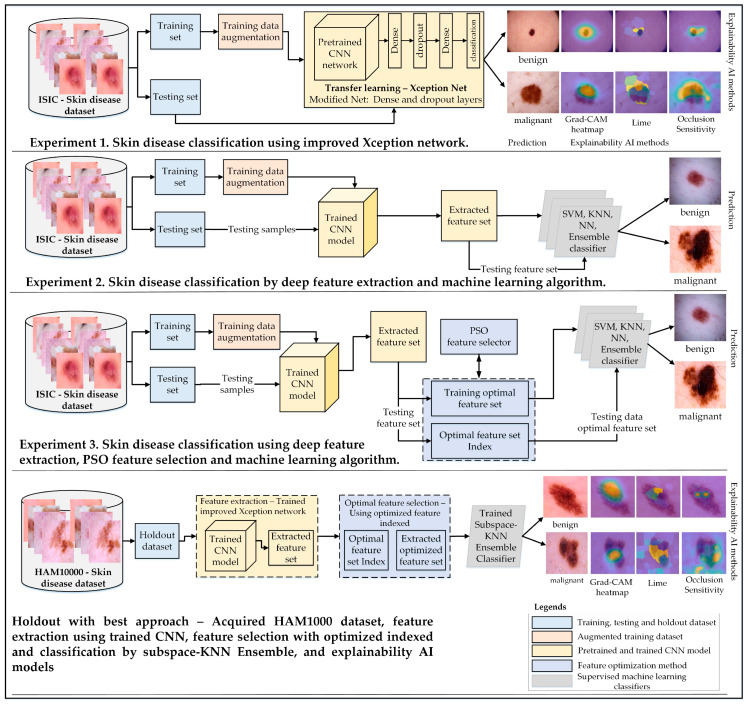
The complete pipeline of the proposed methodology.

The proposed methodology was further validated using the HAM10000 dataset as a holdout set, showcasing its generalization capabilities. Explainable AI techniques such as Grad-CAM, LIME, and Occlusion Sensitivity were employed across all experiments to enhance model interpretability and transparency. Figure 1 provides a comprehensive overview of the experimental setup, including dataset preparation, augmentation, network modification, feature selection, and classification strategies.

### 3.1. Dataset Collection, Preprocessing, and Augmentation

#### 3.1.1. ISIC Skin Cancer: Malignant vs. Benign

The dataset used in this study for skin cancer detection and recognition was obtained from the ISIC 2018 dataset [52], publicly available on Kaggle data repository, and designed specifically for malignant vs. benign skin lesion classification. This dataset comprised a total of 3297 images, with a total memory size of 340 MB. Each image is in RGB format, measuring 224 × 244 pixels, and is categorized into either the benign or malignant class. The benign class encompasses 1800 images, while the malignant class contains 1497 images. To facilitate model training and evaluation, the dataset was meticulously divided into training and testing subsets. The training dataset comprised 1440 benign images and 1197 malignant images, while the testing dataset included 360 benign images and 300 malignant images. To address class imbalances, we applied data augmentation techniques mentioned in Section 2.1, ensuring equivalent distribution of augmented samples. This process generated a balanced dataset of 10,548 images, with 5760 benign and 4788 malignant samples as elaborated in Table 2. This meticulous data collection and division strategy ensured a balanced and comprehensive approach to training and accurately testing deep-learning-based classification models for skin cancer diagnosis and recognition.

#### 3.1.2. Human Against Machine Dataset

The HAM10000 (Human Against Machine with 10,000 images) [53] dataset is openly accessible on Kaggle platform and is extensively utilized for tasks including binary classification, skin lesion segmentation, and benchmarking the performance of human experts and machine-learning models in dermatology. It comprises 10,015 dermoscopic images designed to train and evaluate machine-learning models for skin lesion diagnosis. The original dataset is categorized into seven classes—actinic keratoses and intraepithelial carcinoma (akiec), Basal cell carcinoma (bcc), benign keratosis-like lesions (bkl), dermatofibroma (df), melanoma (mel), melanocytic nevi (nv), and vascular lesions (vasc)—and have been grouped into two broader categories in our research: benign (bkl, df, nv, vasc) and malignant (akiec, bcc, mel). The dataset includes metadata such as patient age, gender, and lesion location and is imbalanced, with common classes like “nv” dominating. The detailed distribution of the samples across these categories is shown in Table 3.

#### 3.1.3. Data Augmentation

The data augmentation [54] strategy employed aimed to augment the dataset’s diversity and robustness to enhance the performance of the model. Initially, the original images were preserved, ensuring a reference point for comparison. Subsequently, three primary augmentation techniques were applied with specific parameter configurations.

Random rotation, ranging from −180 to 180 degrees, introduced variations in object orientations, essential for capturing a broad spectrum of viewpoints and perspectives computed by following mathematical equations.

(1) $$\left[\begin{array}{c} u’\\ v’ \end{array}\right]=\left[\begin{array}{cc} \cos\left(\theta \right) & \;-\sin\left(\theta \right)\\ \sin\left(\theta \right) & \;\cos\left(\theta \right) \end{array}\right]\;\left[\begin{array}{cc} u & {\;Center}_{u}\\ v & {\;Center}_{v} \end{array}\right]+\left[\begin{array}{c} {Center}_{u}\\ {Center}_{v} \end{array}\right]$$

In the image rotation operation, u’ and v’ represent the coordinates of the rotated pixel, while u and v denote the original coordinates of the input image pixel. The rotation angle θ, randomly generated within the range of −180 to 180 degrees, was measured in radians. (${Center}_{u},\;{Center}_{v}$) refer to the coordinates of the rotation center.

Gaussian blur, utilizing a standard deviation of 2, effectively smooths images by diminishing high-frequency noise while retaining critical features, thereby bolstering the model’s resilience to noise and minor variations. The Gaussian blur operation was implemented using the following equation:

(2) $$I_{g\_blur(u,v)}=\sum_{i=-\infty }^{\infty }\sum_{j=-\infty }^{\infty }I\left(u+i,\;v+j\right)\;.\;G(i,j)$$

The value of $I_{g\_blur(u,v)}$ represents the pixel value at coordinates (u,v) in the blurred image. While $I\left(u+i,\;v+j\right)$ denotes the pixel value at coordinates $\left(u+i,\;v+j\right)$ in the original image. G(i,j) represents the Gaussian kernel value at coordinates (i,j), calculated using a standard deviation of 2.

The Gaussian kernel G(i,j) is computed as follows:

(3) $$G\left(i,j\right)=\frac{1}{{2\pi \sigma }^{2}}\;.\;e^{-\frac{i^{2}+j^{2}}{{2\sigma }^{2}}}$$

where σ is the standard deviation, which in this case is 2.

**Figure 2:**
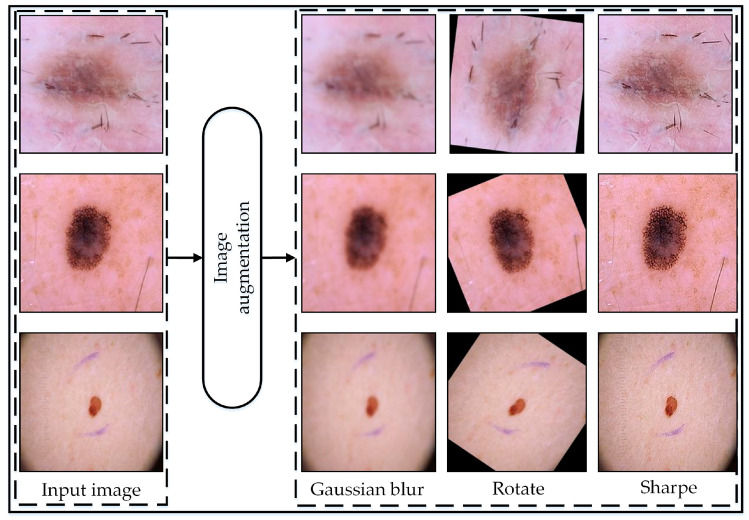
Resulting images of augmentation operation.

Furthermore, image sharpening augmentation was applied to enhance edge sharpness and detail prominence within the image, crucial for accurately capturing fine-grained features and textures. The parameters set for this operation included an ‘Amount’ of 2, indicating a strong sharpening effect, a ‘Radius’ of 1 for enhancing finer details, and a ‘Threshold’ of 0, meaning all pixels will undergo sharpening without a minimum change in intensity requirement. Through the meticulous adjustment of augmentation parameters, the dataset was enriched with a diverse array of visual characteristics, empowering the model to generalize more effectively and achieve superior performance across a spectrum of real-world scenarios. The augmentation procedure was only undertaken to train images to improve the model’s accuracy. Figure 2 illustrates the visual representation of the augmented data, while Table 2 displays the number of images before and after augmentation.

Overall, we considered only a few simple augmentation techniques to minimize computational complexity. We determined that these techniques were sufficient, as they provided a high enough number of samples, which we found adequate based on an analysis of the experimental results.

### 3.2. Transfer Learning

Transfer learning often involves pre-training the network on a large dataset and then fine-tuning it on a specific task allowing the model better generalization and improved performance, especially when limited labeled data are available [55].

In this study, we employed a pre-trained CNN-based model known as Xception [30]. The Xception-Net, developed by Francois Chollet in 2016, is a CNN model derived from Inception architecture consisting of 36 convolutional layers integrating depth-wise separable convolutions for efficiency. The network excels in classification and feature extraction tasks due to its innovative architecture, which incorporates depth-wise separable convolutions. This design reduces the number of parameters while capturing spatial and channel wise dependencies effectively. Xception’s hierarchical feature representation enables it to learn intricate details at different levels of abstraction, contributing to its strong performance in image classification benchmarks. Additionally, its transfer-learning capabilities allow for efficient fine-tuning on specific tasks, making it a preferred choice for various computer vision applications where accuracy and efficiency are paramount.

### 3.3. Modification of the Xception Network Architecture

To train the network for skin cancer classification and feature learning, we modified the architecture by excluding pre-trained layers including the last dense, softmax, and classification layer. Additionally, we froze the weights of the upper layers of the model to follow up the training mechanics of the transfer-learning approach. Then we modified the architecture to include a global average pooling (GAP) layer followed by a fully connected layer with 1024 neurons. This modification enables the network to extract more abstract and high-level features from the input features matrix. Additionally, we employed a dropout layer with a probability score of 0.5 which prevented overfitting by randomly deactivating neurons during training. Finally, the addition of a fully connected layer with 2 neurons for classification allows the network to predict benign and malignant skin cancer detection. In summary, the modifications and learning parameter configuration enhanced the network’s adaptability and performance for skin cancer detection.

After the training process, we utilized a trained model and used it with two different aspects focused on feature importance and skin cancer detection.

### 3.4. Feature Selection Using PSO

In this step, a trained Xception network was utilized to extract a training feature set from a dense layer comprising 1024 neurons, yielding 1024 features per image. The PSO [38] features’ selection method was subsequently applied. Originally proposed by Kennedy and Eberhart in 1995, the PSO algorithm operates by simulating the movement of a group of particles in a search space, with each particle representing a potential solution to the optimization problem. These particles navigate through the search space based on their current position, velocity, and the optimal positions discovered by themselves and their neighbors. A major advantage of this technique is its ability to autonomously determine the optimal feature dimensions, eliminating the need for trial-and-error approaches commonly used in conventional methods. This ensures a more efficient and robust feature selection process.

In the process of optimizing feature selection for robust feature selection, the k-Nearest Neighbors (KNN) classifier serves as the fitness function within the PSO algorithm, where k is 5. This fitness function evaluates the performance of selected image features in classifying skin lesions. Specifically, the KNN classifier is trained on the subset of features chosen by the PSO algorithm and subsequently measures its accuracy in predicting the labels (benign or malignant) of skin lesions. The fitness value calculated by the KNN classifier serves as a metric for assessing the effectiveness of the selected features in distinguishing between benign and malignant lesions. Through this iterative evaluation process, the PSO algorithm dynamically adjusts the selection of features to optimize the classification performance of the KNN classifier. In Figure S1, located in Section S3.4 of the Supplementary Material, the performance of learning and selection stages of features have been shown in terms of fitness values across iterations. Ultimately, the collaborative effort between the PSO algorithm and the KNN fitness function aimed to identify the most informative features for accurate skin cancer detection, thereby facilitating early diagnosis and treatment.

The PSO learning process begins with the definition of key parameters, each accompanied by its explanation, as detailed in Table 4.

After the PSO operation was concluded, the optimal feature set comprised 508 features for individual samples. The total feature dimension is represented by a matrix of size 10,548-by-508. These selected features have been utilized for training and classification within a machine-learning model for skin disease detection, as elaborated upon in subsequent sections.

### 3.5. Classification

Following the feature selection step, we conducted a two-way classification. Initially, we performed classification using a trained CNN model with a softmax function. Subsequently, we employed various machine-learning classifiers, each with their respective base function. These classifiers encompass Linear SVM (L-SVM), Quadratic SVM (Q-SVM), Cubic SVM (C-SVM), Fine Gaussian SVM (FG-SVM), Medium Gaussian SVM (MG-SVM), Coarse Gaussian SVM (CG-SVM), Fine KNN (F-KNN), Medium KNN (M-KNN), Coarse KNN (C-KNN), Cosine KNN (Cos-KNN), Weighted KNN (W-KNN), Boosted Trees Ensemble (BT-Ensemble), Bagged Trees Ensemble (BT-Ensemble), Sub-space Discriminant Ensemble (SD-Ensemble), Subspace KNN Ensemble (SK-Ensemble), and RUSBoosted Trees Ensemble (RBT-Ensemble). These classifiers were utilized for both training and validation, employing a 5-fold cross-validation process.

## 4. Results

We begin the results section by evaluating multiple pretrained CNN models using the same transfer learning setup over five epochs to identify the most suitable model for our study. This initial step was critical to ensure optimal CNN model selection. Among the tested models, including InceptionV3, MobileNet, and EfficientNet, Xception emerged as the best-performing architecture based on its balance of computational efficiency and classification accuracy. The detailed comparison of these models, including their accuracies and parameter complexities, is provided in Table S1 under Supplementary Section S4.2. This selection formed the foundation for the subsequent experiments, where we further performed the Xception network-based experiments for robust skin cancer classification.

Overall, we employed transfer learning techniques, utilizing an enhanced Xception network for both features extraction and classification tasks. Subsequently, we applied features selection algorithms to identify a subset of discriminative features. These selected features were then utilized to train SVM, KNN, and Ensemble classifiers, allowing the evaluation of the system’s performance comprehensively.

All experiments described were implemented and executed using MATLAB 2023b (Version 23.2) software on a Windows operating system. The system was equipped with an Intel Core i7 10th generation processor with 8GB of memory. Additionally, an NVIDIA RTX 2060 GPU with 6GB of dedicated RAM was utilized for accelerated computations.

The proposed methodology utilized the ISIC-2018 dataset consisting of 11,208 dermoscopy images showing various skin illnesses. The dataset was predefined into training and testing sets. Further, training samples of the dataset were separated by K-fold at runtime by maintaining an equitable 70:30 ratio into training and validation, respectively.

**Figure 3:**
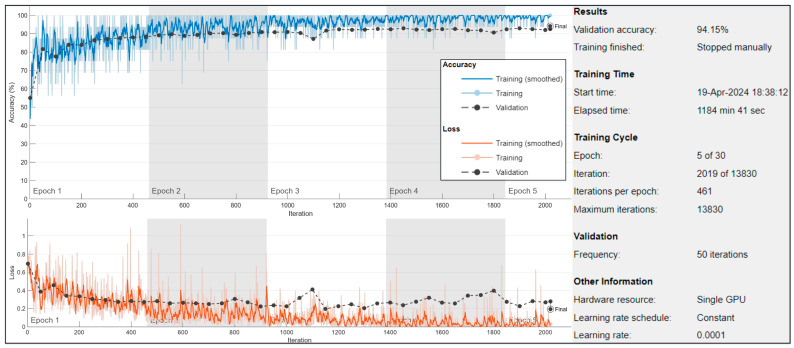
Training and validation accuracy (top) and loss (bottom) curves over iterations during the training process of the proposed model. The gray bars indicate specific epochs of interest, highlighting regions where the training and validation metrics stabilized or showed notable changes. Additional training details, such as elapsed time, learning rate, and hardware resources, are provided for context.

On training data, different types of augmentation were also implemented, named rotation, blurred, and sharpening in the x–y axis (briefly discussed in Section 3.1.3). Further, the Adam optimizer [56] was utilized for optimization, with training execution set to GPU to leverage parallel processing. Mini-batch size was defined as 16, determining the number of samples processed together in each iteration. The training continued for a maximum of 30 epochs, with an initial learning rate of 1 × 10^−4^. Data shuffling occurred before each epoch to prevent overfitting. Validation data, used to evaluate model performance, was provided every 50 mini-batches. The verbose output was suppressed, and training progress plots were generated to monitor performance throughout the training process, as shown in Figure 3.

### Performance Evaluation Metrics

To assess the classification results of the proposed technique, this study employed various performance assessment metrics, including accuracy (Acc), sensitivity (Sen), specificity (Spe), precision (Pre), and F1-Score in Seq 1 to 5 in the Supplementary Materials under Section S4.1.

Overall, we carried out three experiments to assess the effectiveness of our proposed methodology for skin cancer detection. We employed a transfer-learning approach and machine-learning algorithms in two experiments: one with optimal feature selection and one without. Our aim was to highlight the significance of optimal feature selection in achieving higher accuracy scores while reducing computational costs and training time. The overall explanation of the conducted experiments is mentioned below.

1. Experiment # 1: (skin cancer classification by improved Xception network)

In the first experiment, we modified the pretrained Xception model by incorporating global average pooling layers and several dense layers with varying neuron configurations to enhance its capability for transfer learning. This modification allowed the network to better adapt to the binary classification task of distinguishing between benign and malignant skin lesions. Training was focused on the newly introduced layers while freezing the pretrained layers to preserve the general features learned from the original dataset. The model training was performed for five epochs, during which, the model reached its optimal learning stage, and training was manually stopped to avoid overfitting.

**Figure 4:**
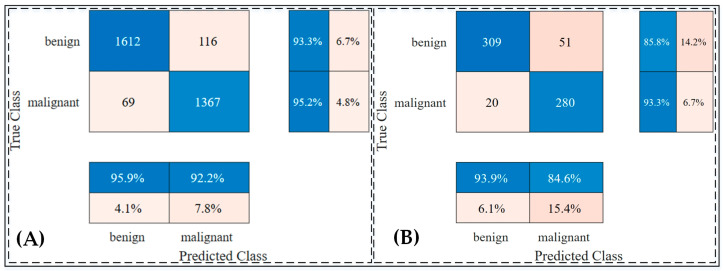
Confusion matrix of improved Xception network: (**A**) confusion matrix on validation dataset; (**B**) confusion matrix on testing dataset.

The trained model was then evaluated on a test set comprising 660 unseen images to assess its performance on independent data. The results demonstrated an accuracy rate of 94.15% on the validation dataset and 89.24% on the testing dataset, showcasing the model’s generalization ability. Figure 4 provides a detailed visualization of the distribution of positive and negative predictions using confusion matrices for both the validation and testing datasets. Furthermore, an ablation study was conducted to validate the effectiveness of the transfer learning approach, confirming that the chosen architecture and training strategy were optimal. Detailed findings from this study are provided in Table S2 and Figures S2, S3, S4, and S5 under Section S4.3 of the Supplementary Materials.

2. Experiment # 2: (skin cancer classification by robust feature extracted and machine-learning classifiers)

**Figure 5:**
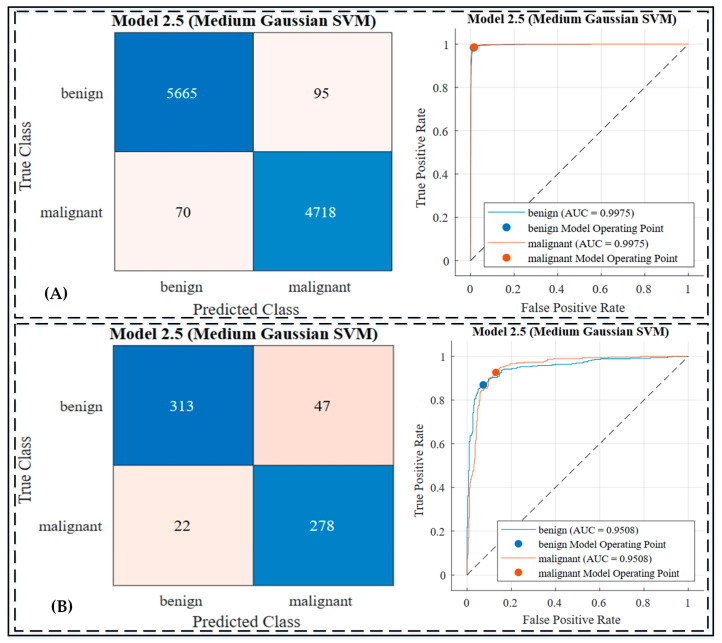
Confusion matrix and ROC curve of Experiment 2 by Medium Gaussian SVM classifier: (**A**) confusion matrix and ROC curve on training dataset; (**B**) confusion matrix and ROC curve on testing dataset. Additionally, the dashed line in the ROC curve represents the reference line for random classification (AUC = 0.5).

In the second experiment, the extracted features from the Xception model were used to train various machine-learning classifiers to evaluate their performance in classifying skin lesions as benign or malignant, as shown in Figure 5, Figures S2 and S3 and Table 5. Among the SVM classifiers, the Linear SVM (L-SVM) achieved an accuracy of 89.2% on the testing dataset, with sensitivity and specificity values of 92.8% and 85.4%, respectively. The Cubic SVM (C-SVM) also exhibited comparable results, achieving a testing accuracy of 89.2%, with sensitivity and specificity of 92.6% and 85.4%, respectively.

In the KNN group, as shown in Figure S2, the Cosine KNN (Cos-KNN) model outperformed others, achieving a testing accuracy of 89.4%, a sensitivity of 95.0%, and a specificity of 84.0%. The Weighted KNN (W-KNN) also showed robust performance, with testing accuracy, sensitivity, and specificity values of 89.1%, 94.7%, and 83.7%, respectively.

The ensemble classifiers provided notable results, as displayed in Figure S3, with the Boosted Trees (BT-Ensemble) classifier achieving a testing accuracy of 89.1%, a sensitivity of 93.1%, and a specificity of 84.9%. Similarly, the Bagged Trees Ensemble (BAGT-Ensemble) achieved an accuracy of 89.1%, a sensitivity of 93.4%, and a specificity of 85.8%.

Across all classifiers, the Medium Gaussian SVM (MG-SVM) demonstrated one of the highest performances, achieving a testing accuracy of 89.5%, a sensitivity of 93.4%, and a specificity of 85.5%. The confusion matrices and ROC curves, illustrated in Figure 5, highlight the effectiveness of these classifiers in distinguishing between benign and malignant lesions. These results underscore the capability of integrating extracted features with robust machine-learning classifiers to achieve high classification performance.

3. Experiment # 3: (skin cancer classification by selective feature set and machine-learning classifiers)

**Figure 6:**
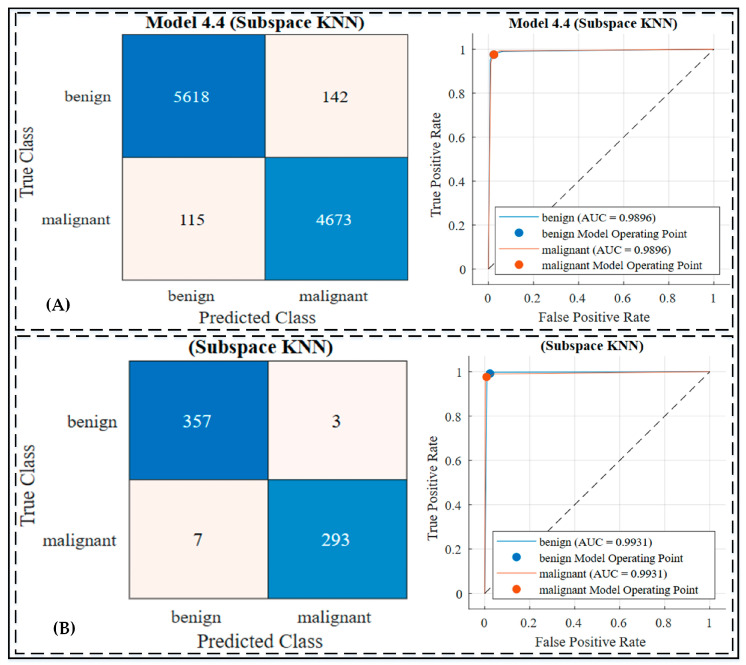
Confusion matrix and ROC curve of Experiment 3 by Ensemble Subspace KNN classifier: (**A**) confusion matrix and ROC curve on training dataset; (**B**) confusion matrix and ROC curve on testing dataset. Additionally, the dashed line in the ROC curve represents the reference line for random classification (AUC = 0.5).

In Experiment 3, the integration of deep feature extraction, Particle Swarm Optimization (PSO)-based feature selection, and machine-learning classifiers was employed to evaluate their impact on skin cancer classification. Deep features extracted from the transfer-learned Xception network were reduced from 1024 to 504 dimensions using the PSO algorithm, ensuring computational efficiency without compromising accuracy. Using this reduced feature set, multiple machine-learning classifiers were trained and evaluated using a 5-fold cross-validation strategy as results are displayed in Table 6 and Figure 6 and Figures S4 and S5. Among the classifiers, Cubic SVM achieved the highest testing accuracy of 98.0%, with a sensitivity of 98.2%, a specificity of 97.5%, and an F1 score of 98.0%, as shown in Figure S4. The Fine-KNN classifier performed comparably with a testing accuracy of 98.2% and demonstrated robust sensitivity (98.0%) and specificity (98.3%), as displayed in Figure S5.

The ensemble classifiers also showcased high performance, with the Subspace KNN ensemble achieving the best results across all metrics, including an accuracy of 98.5%, a sensitivity of 98.0%, a specificity of 98.9%, a precision of 99.1%, and an F1 score of 98.6%. These results highlight the advantage of combining optimized feature sets with advanced machine-learning models. Computational metrics, including total costs, training times, and prediction speeds, were also evaluated, with the Subspace KNN ensemble achieving optimal efficiency and predictive power. Figure 6 illustrate the confusion matrices and ROC curves for the top-performing classifiers, which demonstrated excellent discriminative capabilities, as evidenced by their AUC values exceeding 0.99.

Holdout results based on all Experiments

**Figure 7:**
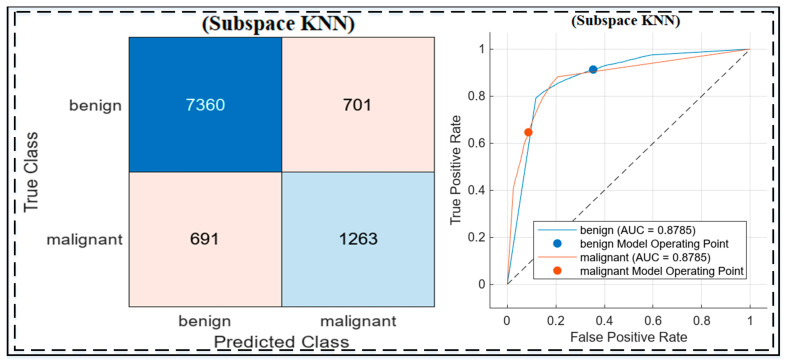
Confusion matrix and ROC curve for the Subspace KNN classifier on the HAM10000 dataset, showing classification performance with an AUC of 0.8785 for both benign and malignant classes. The confusion matrix highlights true positives, false positives, and misclassifications, while the ROC curve demonstrates the model’s discriminative ability. Additionally, the dashed line in the ROC curve represents the reference line for random classification (AUC = 0.5).

The holdout experiments on the HAM10000 dataset evaluated the performance of the three approaches, highlighting the strengths of the proposed methodology, as shown in Figure 7 and Table 7. The baseline Xception model (Experiment 1) achieved high sensitivity (94.2%) but struggled with specificity (49.8%) and overall accuracy (80.3%). Adding a Gaussian SVM in Experiment 2 slightly improved accuracy (81.05%) and specificity (51.3%) but still fell short of optimal performance. The best results were obtained in Experiment 3, where Xception was combined with Particle Swarm Optimization (PSO) for feature selection and was classified using Subspace KNN. This approach achieved an accuracy of 86.1%, a sensitivity of 91.42%, and a specificity of 64.31%, demonstrating a better balance in distinguishing benign and malignant lesions. The integration of PSO and Subspace KNN significantly enhanced feature discrimination and classification performance, as illustrated by the confusion matrix and ROC curve. These results validate the robustness and generalization capability of the proposed methodology in handling imbalanced datasets like HAM10000.

## 5. Discussion

1. Experiment 1: Transfer-Learned Xception with Softmax Classifier

In the first experiment, the pretrained Xception model was modified with a global average pooling layer and multiple dense layers to enhance its feature representation. This experiment achieved a training accuracy of 98.4% and a testing accuracy of 89.7% on the ISIC 2018 dataset. The results highlighted the capability of the Xception network to differentiate between benign and malignant skin lesions effectively. As shown in Table 8, the model performed well in sensitivity (93.9%) but exhibited moderate specificity (84.6%), indicating its strength in detecting malignant cases but room for improvement in distinguishing benign cases.

2. Experiment 2: Deep Features with Machine-Learning Classifiers

The second experiment leveraged deep feature extraction from the transfer-learned Xception network and evaluated various machine-learning classifiers. Among the classifiers, the Medium Gaussian SVM (MG-SVM) achieved the best performance with a testing accuracy of 89.6%, a sensitivity of 93.4%, and a specificity of 85.5%. These results emphasize its ability to detect malignant lesions effectively, which is critical for early intervention. In the KNN group, the Cosine KNN (Cos-KNN) demonstrated strong sensitivity (95.0%), further minimizing false negatives. Ensemble classifiers, such as Boosted Trees Ensemble, provided balanced performance, achieving a testing accuracy of 89.1% and a sensitivity of 93.1%. Figure 5 illustrates the confusion matrices and ROC curves for the top classifier, which validate their ability to distinguish between classes effectively. While the performance was slightly lower than Experiment 3, the results highlighted the value of machine-learning classifiers in enhancing the predictive power of deep feature extraction.

3. Experiment 3: Feature Selection and Subspace KNN Ensemble

The third experiment integrated deep feature extraction, PSO-based feature selection, and advanced machine-learning classifiers to optimize skin cancer classification. Using the PSO algorithm, feature dimensions were reduced from 1024 to 504, significantly improving computational efficiency without sacrificing accuracy. The Subspace KNN ensemble emerged as the top-performing model, achieving a testing accuracy of 98.5%, a sensitivity of 98.1%, a specificity of 98.9%, and a precision of 99.1%. These results validate the superiority of the optimized pipeline in handling complex decision boundaries and achieving high predictive accuracy. Figure 6 showcases the confusion matrices and ROC curves for the Subspace KNN classifier, demonstrating minimal false positives and false negatives. Compared with previous methodologies, such as DenseNet201 and SCSO-ResNet50-EHS-CNN, the proposed approach outperformed them on the ISIC 2018 dataset in all metrics, underscoring the importance of feature selection and ensemble learning.

4. Comparison with Previous Works

The comparison with previous works highlights the efficacy and limitations of the proposed methodology when benchmarked on both the HAM10000 and ISIC-2018 datasets, as illustrated in Table 9. On the ISIC-2018 dataset, the proposed methodology demonstrated state-of-the-art performance, achieving 98.5% accuracy, 98.1% sensitivity, and 98.9% specificity with the Xception + PSO + Subspace KNN pipeline (Experiment 3). This significantly outperformed prior studies, such as [39] and Akilandasowmya, Nirmaladevi, Suganthi and Aishwariya [42], who achieved 92% accuracy using DenseNet201 and SCSO-ResNet50-EHS-CNN, respectively. Although Akilandasowmya, Nirmaladevi, Suganthi and Aishwariya [42] achieved a higher sensitivity of 93.9%, their specificity of 85.5% was notably lower compared with the proposed method’s superior balance of sensitivity and specificity. Additionally, Saha, Joy and Majumder [49], integrating ViT and MobileNet with segmentation techniques on ISIC 2019, achieved an accuracy of 91.2%, with an approximate sensitivity and specificity of 93% and 90%, respectively. While their results show competitive performance, the proposed method still leads in accuracy and specificity, crucial for reducing false positives and enhancing diagnostic reliability in clinical settings. This underscores the superiority of the proposed pipeline, which combines deep feature extraction, PSO-based feature optimization, and ensemble classifiers to provide a robust diagnostic tool for skin cancer detection, especially well-suited for settings that demand high accuracy and detailed lesion analysis. Further, earlier experiments in this study, such as Experiment 1 (standalone Xception) and Experiment 2 (Xception + Gaussian SVM), achieved accuracies of 89.7% and 89.6%, respectively, further highlighting the incremental benefits of feature selection and ensemble learning. The proposed approach’s ability to optimize performance through PSO-based feature reduction and ensemble classifiers emphasizes its potential for clinical application, providing accurate and reliable predictions for both benign and malignant skin lesions.

On the HAM10000 dataset, the proposed Xception + PSO + Subspace KNN method achieved an accuracy of 86.1%, a sensitivity of 91.42%, and a specificity of 64.31%. This performance highlights a strong ability to detect malignant cases effectively, as evidenced by its high sensitivity, which surpasses Raju, Hemalatha, Goli, Yuvananda, Karthik and Krishna [40] (84% sensitivity) who employed CGAN with ensemble models, and is comparable to the sensitivity (~92%) reported by Ahmad, Alsulami and Alqurashi [48] using ViT and CNNs. However, the lower specificity of the proposed approach indicates room for improvement in reducing false positives. In comparison, Ali, Miah, Haque, Rahman and Islam [41] demonstrated superior balance, achieving 93% accuracy, 91% sensitivity, and 94% specificity with their custom CNN. It is worth noting that the proposed method evaluated HAM10000 as a completely unseen holdout set, unlike other studies that used it for training and validation. This underscores the robustness and generalization potential of our methodology, while also identifying the need to enhance specificity for better clinical alignment in future iterations.

## 6. Visual Representation of Model Attention Using Grad-CAM, LIME, and Occlusion Sensitivity

Gradient-Weighted Class Activation Mapping (Grad-CAM) [62], Local Interpretable Model-Agnostic Explanations (LIME) [51], and Occlusion Sensitivity [63] are powerful tools used to visualize and interpret deep-learning models’ attention to regions of interest (ROI) within images. These techniques help to identify the areas in an image that contribute the most to the model’s decision-making process, thereby enhancing the interpretability of predictions.

The visualization process involves passing an input image through the pretrained Xception model, predicting the label, and mapping the attention regions. Grad-CAM highlights the pixels of the last convolutional layer corresponding to significant features, with red areas indicating regions of highest importance. LIME generates explanations by perturbing image pixels and evaluating their impact on the predictions, providing interpretable feature importance maps. Occlusion Sensitivity further validates the predictions by systematically masking parts of the image and observing changes in the output.

**Figure 8:**
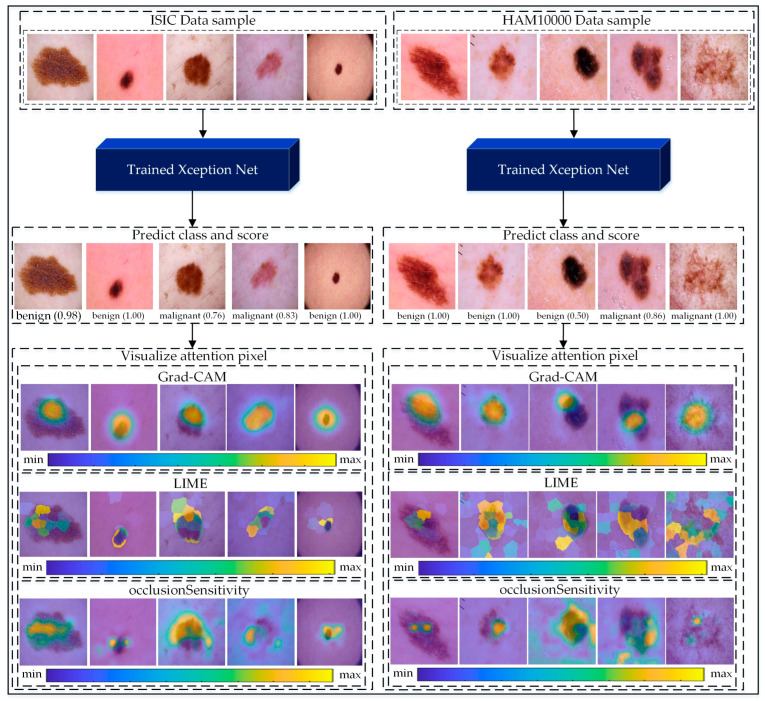
Visualization of the proposed Xception-based pipeline applied to ISIC and HAM10000 datasets for skin cancer classification. Input images are classified as benign or malignant with confidence scores. Grad-CAM highlights critical regions, LIME provides pixel-level interpretations, and Occlusion Sensitivity validates predictions, enhancing model transparency for clinical applications. Additionally, the color legend bars indicate the intensity of contribution, with “min” and “max” representing low to high importance, enhancing the model’s transparency and interpretability for clinical applications.

As illustrated in Figure 8, we applied these methods to samples from the ISIC and HAM10000 datasets. For benign and malignant cases, Grad-CAM heatmaps show distinct areas of focus, with red indicating critical lesion regions. LIME explanations overlay relevant pixel clusters, providing a fine-grained interpretation. Occlusion Sensitivity complements these methods by visualizing the impact of occluding different regions on prediction confidence. Together, these approaches reveal the decision-making process of the Xception model, enhancing its transparency and reliability.

The proposed methodology makes the approach suitable for deployment in resource-constrained settings. The integration of explainable AI techniques, such as Grad-CAM, LIME, and Occlusion Sensitivity, provided insights into the model’s decision-making process, fostering trust among medical practitioners by highlighting critical regions in skin lesions. These results have significant implications for clinical practice, including improving diagnostic accuracy, reducing misdiagnoses, and supporting early treatment through reliable, interpretable tools. The methodology also aligns with clinical workflows by acting as a decision-support system, ensuring that AI complements rather than replaces human expertise. Its scalability and transparency make it a promising tool for telemedicine and rural healthcare, potentially democratizing access to advanced diagnostic technologies. Future research can build upon these findings by exploring diverse datasets, expanding the approach to other skin diseases, and further refining interpretability techniques to enhance its clinical applicability.

Further, different XAI techniques suit different clinical contexts. For example, Grad-CAM is better suited to providing an intuitive and quick overview for decision-making, while LIME and Occlusion Sensitivity are more appropriate for detailed validation and in-depth analysis of specific cases. This versatility ensures that the explainability framework aligns with varying diagnostic behaviors and preferences of medical practitioners.

## 7. Conclusions

This study explored the effectiveness of a CNN-based Xception network, deep feature extraction, and optimal feature selection for skin cancer detection, with the primary goal of improving classification accuracy and computational efficiency. Three distinct approaches were examined: classification using transfer learning with the Xception network, deep feature extraction paired with machine-learning classifiers, and feature extraction combined with Particle Swarm Optimization (PSO) for dimensionality reduction and subsequent classification. The integration of data augmentation techniques, including image rotation, Gaussian blur, and sharpening, allowed for the preparation of a robust training dataset using the ISIC and HAM10000 dataset as a holdout. Our results demonstrated the highest testing accuracy of 98.5% on the ISIC dataset by combining Xception-based feature extraction, PSO-based feature selection, and Subspace KNN classifiers. On the HAM10000 holdout dataset, the methodology achieved an accuracy of 87.1%. The Grad-CAM, LIME, and Occlusion Sensitivity explainable-AI technique were employed to visualize the model’s attention, enhancing interpretability and providing insights into the classification process.

In the future, we aim to validate the model’s adaptability to diverse skin types and imaging conditions by using larger and more heterogeneous datasets. Efforts will focus on enhancing data diversity through GANs, expanding the methodology to cover other dermatological diseases, and improving interpretability with advanced explainable AI techniques. Collaboration with dermatologists will also be prioritized to evaluate the model’s practical utility as a clinical decision support tool, ensuring its outputs are interpretable, relevant, and seamlessly integrated into real-world clinical workflows.

**Scientific Insights:** This research not only aims for a technically robust AI pipeline but also contributes significant scientific insights that enhance the understanding of AI’s role in medical imaging and its real-world applications.

- **Enhanced Diagnostic Accuracy:** By integrating transfer learning with PSO, this study achieves high classification accuracy while reducing computational costs, fulfilling the aim of building an efficient diagnostic tool.
- **Adaptability Across Clinical Datasets:** The generalization of the proposed pipeline on ISIC 2018 and HAM10000 datasets demonstrates its ability to address diverse clinical and demographic challenges.
- **Interpretability for Clinical Decision Support:** The inclusion of Grad-CAM, LIME, and Occlusion Sensitivity aligns with this paper’s focus on explainability, bridging the gap between AI predictions and clinical trust.
- **Scalability in Resource-Limited Settings:** The computational efficiency achieved through dimensionality reduction ensures the pipeline’s suitability for real-world deployment, particularly in resource-constrained environments.
- **Framework for Expanding Medical Image Applications:** The interdisciplinary impact of the optimized pipeline serves as a foundation for diagnosing other medical conditions, aligning with the broader aim of enhancing AI’s role in healthcare.

## Figures and Tables

**Table 2:** Number of training samples before and after augmentation.

Classes	Before Augmentation	After Augmentation
Benign	1440	5760
Malignant	1197	4788
total	2637	10,548

**Table 3:** Detailed distribution of the HAM10000 dataset, showing the original seven skin lesion classes, their respective number of samples, and their grouping into two merged categories (benign and malignant) for binary classification.

	Classes	No. of Samples	Merged Classes	No. of Samples
HAM1000	bkl	1099	benign	8061
	df	115		
	nv	6705		
	Vasc	142		
	Akiec	327	malignant	1954
	Bcc	514		
	mel	1113		

**Table 4:** Initial learning parameters of the PSO algorithm.

Parameter	Initial Value	Description
T	100	Maximum number of iterations
C1	2.5	Cognitive factor
C2	2.5	Social factor
Vmax	7	Maximum velocity
Wmax	0.95	Maximum bound on inertia weight
Wmin	0.35	Minimum bound on inertia weight

**Table 5:** Performance Metrics, Computational Costs, Training Times, and Prediction Speeds of Machine-Learning Classifiers for Skin Cancer Classification Using Features Extracted from the Transfer-Learned Xception Network.

Classifier	Acc (%)	Sen (%)	Spe (%)	Pre (%)	F1 (%)	Total Cost	Training Time (s)	Prediction Speed ~(obs/s)
L-SVM [57]	98.5/89.2	98.8/92.8	98.9/85.4	98.2/86.9	98.5/89.8	163/71	76.868	3900
Q-SVM [57]	98.4/89.2	98.8/92.8	98.0/85.4	98.3/86.9	98.5/89.8	164/71	76.142	3600
C-SVM [57]	98.5/89.2	98.8/92.6	98.0/85.4	98.3/86.9	98.6/89.6	162/72	167.31	1100
FG-SVM [57]	87.6/75.2	82.2/72.0	97.7/81.4	98.5/88.9	89.6/79.6	1305/164	1069.8	100
MG-SVM [57]	**98.4/89.5**	**98.7/93.4**	**98.0/85.5**	**98.3/86.9**	**98.5/90.0**	**165/69**	**166.2**	**1300**
CG-SVM [57]	98.2/89.4	98.4/93.4	97.9/85.2	98.3/86.6	98.3/89.9	188/70	230.94	600
F-KNN [58]	98.2/88.6	98.3/92.5	98.0/84.6	98.3/86.1	98.3/89.2	190/75	1108.4	51
M-KNN [58]	98.2/88.9	98.8/94.1	97.4/83.8	97.8/85.0	98.3/89.3	187/73	933.71	50
C-KNN [58]	98.2/88.8	98.4/93.6	97.8/84.0	98.2/85.2	98.3/89.2	190/74	1146.8	45
Cos-KNN [58]	**98.3/89.4**	**98.8/95.0**	**97.6/84.0**	**98.0/85.0**	**98.4/89.7**	**179/70**	**1286.7**	**43**
W-KNN [58]	98.2/89.1	99.0/94.7	97.2/83.7	97.6/84.7	98.3/89.4	187/72	1497.3	93
BT-Ensemble [59]	**98.3/89.1**	**98.5/93.1**	**97.9/84.9**	**98.2/86.3**	**98.4/89.6**	**181/72**	**2190.2**	**4600**
BAGT-Ensemble [59]	98.3/89.1	98.4/93.4	98.1/85.8	98.3/87.2	98.4/90.2	183/68	3515.8	8600
SD-Ensemble [60]	98.3/88.3	98.7/91.7	97.7/84.7	98.0/86.3	98.4/88.9	181/77	1881.9	360
RUSBoosted Trees [61]	98.1/88.8	98.4/93.6	97.7/84.0	98.1/85.2	98.2/89.2	198/74	2526.8	6900

The bold values in the table highlight the best-performing metrics within each group of classifier models. For instance, MG-SVM has performed better among the SVM classifiers, Cos-KNN has shown superior performance among the KNN classifiers, and BT-Ensemble has achieved the highest metrics among the bagged and tree-based classifiers.

**Table 6:** The classification results of Experiment 3 with respect to multiple machine-learning classifier with the use of 504 selective features.

**Classifier**	**Acc (%)**	**Sen (%)**	**Spe (%)**	**Pre (%)**	**F1 (%)**	**Total Cost**	**Training Time (s)**	**Prediction Speed** **~(obs/s)**
L-SVM [57]	97.8/97.3	98.3/96.7	97.1/97.9	97.5/98.3	97.9/97.5	237/10	72.871	7600
Q-SVM [57]	97.8/97.6	98.3/97.2	97.2/97.9	97.6/98.3	98.0/97.7	230/16	72.151	7500
C-SVM [57]	**97.9/98.0**	**98.2/97.5**	**97.4/98.3**	**97.8/98.6**	**98.0/98.0**	**243/13**	**67.656**	**4900**
FG-SVM [57]	86.4/87.4	81.1/81.8	96.5/98.2	97.8/98.8	88.7/89.5	1436/83	1085.1	160
MG-SVM [57]	97.8/97.6	98.4/96.7	97.0/98.6	97.5/98.8	98.0/97.8	236/16	81.589	4400
CG-SVM [57]	97.7/97.4	98.1/96.2	97.1/98.9	97.5/99.1	97.8/97.6	244/17	122.22	1200
F-KNN [58]	**97.3/98.2**	**97.7/98.0**	**96.8/98.3**	**97.3/98.6**	**97.5/98.3**	**283/12**	**376.01**	**130**
M-KNN [58]	97.6/97.7	98.4/98.3	96.6/97.0	97.1/97.5	97.8/97.9	247/15	403.3	140
C-KNN [58]	97.6/97.7	98.3/97.2	96.7/98.3	97.2/98.6	97.6/97.9	246/15	396.93	140
Cos-KNN [58]	97.6/97.8	98.5/97.7	96.6/97.9	97.1/98.3	97.8/98.8	243/14	542.63	100
W-KNN [58]	97.7/97.7	98.8/98.3	96.4/97.0	97.0/97.5	97.9/97.9	246/15	615.33	190
BT-Ensemble [59]	97.6/97.8	97.9/97.0	97.0/98.9	97.5/99.1	97.7/98.0	250/14	797.69	11,000
BAGT-Ensemble [59]	97.7/97.4	98.0/96.7	97.2/98.2	97.7/98.6	97.9/97.6	253/13	1427.2	14,000
SD-Ensemble [60]	97.7/97.7	98.3/97.0	96.9/98.6	97.4/98.8	97.8/97.9	248/15	720.25	1000
RUSBoosted Trees [61]	**97.6/98.5**	**98.2/98.0**	**97.2/98.9**	**97.6/99.1**	**97.9/98.6**	**257/10**	**2005.4**	**34**

The bold values in the table represent the best-performing metrics within each group of classifier models. Specifically, C-SVM has shown the highest performance among the SVM classifiers, F-KNN has excelled among the KNN classifiers, and RUSBoosted Trees has achieved the best results among the bagged and tree-based classifiers.

**Table 7:** Performance metrics for the best experiment SK-Ensemble classifier on the HAM10000 dataset, including accuracy, sensitivity, specificity, precision, and F1 score. The results highlight the model’s high sensitivity in detecting malignant lesions but lower specificity for benign cases.

Classifier	Acc (%)	Sen (%)	Spe (%)	Pre (%)	F1 (%)
Xception (Experiment 1)	80.3	94.2	49.8	80.5	86.8
Xception + Gaussian SVM (Experiment 2)	81.05	89.1	51.3	87.1	88.0
**Xception + PSO + Subspace KNN (Experiment 3)**	**86.1**	**91.42**	**64.31**	**91.3**	**91.3**

The bold values in the table represent the best-performing metrics across all experiments. Specifically, Xception + PSO + Subspace KNN (Experiment 3) has achieved the highest performance, demonstrating superior accuracy, sensitivity, specificity, precision, and F1-score compared to the other approaches.

**Table 8:** The best three performed models’ performances in three experiments where Acc, Sen, Spe, Pre, and F1 means Accuracy, Sensitivity, Specificity, Precision, and F1-Score, respectively.

Exp #	Classifier	Validation					Test				
		Acc	Sen	Spe	Pre	F1	Acc	Sen	Spe	Pre	F1
Exp1	Softmax	94.2	95.9	92.2	93.3	94.6	89.7	93.9	84.6	85.8	89.7
Exp2	Medium Gaussian-SVM	98.6	98.8	98.0	98.4	98.6	89.6	93.4	85.5	86.9	90.0
Exp3	Subspace-KNN	97.6	97.9	97.1	97.5	97.8	98.5	98.1	98.9	99.2	98.6

**Table 9:** Comparison of various methodologies for skin cancer classification across HAM10000 and ISIC datasets, showcasing accuracy, sensitivity, and specificity metrics for different pipelines, including the proposed Xception-based approaches.

Methodology	Pipeline	Dataset	Accuracy (%)	Sensitivity (%)	Specificity (%)
Raju, Hemalatha, Goli, Yuvananda, Karthik and Krishna [40]	CGAN + Ensembled models	HAM10000	86	84	88
Ali, Miah, Haque, Rahman and Islam [41]	Custom CNN	HAM10000	93	91	94
Ahmad, Alsulami and Alqurashi [48]	Skin lesion classifi-cation using ViT and CNNs	HAM10000	~90	~92	~89
Proposed Method	Xception + PSO + Subspace KNN (Experiment 3)	HAM10000	86.1	91.42	64.31
Al-Rasheed, Ksibi, Ayadi, Alzahrani, Zakariah and Ali Hakami [39]	DenseNet201	ISIC 2019	92	90	93
Akilandasowmya, Nirmaladevi, Suganthi and Aishwariya [42]	SCSO-ResNet50-EHS-CNN	ISIC 2019	92	93.9	85.5
Saha, Joy and Majumder [49]	Integration of ViT and MobileNet with segmentation techniques	ISIC 2019	91.2	~93	~90
Proposed Method	Xception (Experiment 1)	ISIC 2018	89.7	93.9	84.6
Proposed Method	Xception + Gaussian SVM (Experiment 2)	ISIC 2018	89.6	93.4	85.5
Proposed Method	Xception + PSO + Subspace KNN (Experiment 3)	ISIC 2018	98.5	98.1	98.9

## Data Availability

We have utilized publicly available datasets and we referenced them in this manuscript.

## Supplementary Materials

The following supporting information can be downloaded at: https://www.mdpi.com/article/10.3390/jimaging10120332/s1, Figure S1: The Evolution of fitness values throughout the PSO learning.; Table S1: Performance comparison of various pre-trained CNN models (EfficientNet, Inception-V3, MobileNet, and Xception) for skin cancer classification, including metrics (Acc, Sen, Spe, Pre, F1) for validation and testing datasets, along with the number of layers.; Table S2: Ablation study results for freezing different percentages of Xception’s 173 layers, with metrics (Acc, Sen, Spe, Pre, F1) for validation and testing datasets. The 100% freezing configuration was chosen for its comparable performance and reduced computational cost.; Figure S2: Confusion matrix and ROC curve of experiment 2 by Cosine-KNN classifier: (A) Confusion matrix and ROC curve on training dataset (B) confusion matrix and ROC curve on testing dataset. Additionally, the dashed line in the ROC curve represents the reference line for random classification (AUC = 0.5).; Figure S3: Confusion matrix and ROC curve of experiment 2 by Ensemble-Boosted tree classifier: (A) Confusion matrix and ROC curve on training dataset (B) confusion matrix and ROC curve on testing dataset. Additionally, the dashed line in the ROC curve represents the reference line for random classification (AUC = 0.5).; Figure S4: Confusion matrix and ROC curve of experiment 3 by Cubic SVM classifier: (A) Confusion matrix and ROC curve on training dataset (B) confusion matrix and ROC curve on testing dataset. Additionally, the dashed line in the ROC curve represents the reference line for random classification (AUC = 0.5).; Figure S5: Confusion matrix and ROC curve of experiment 3 by Fine-KNN classifier: (A) Confusion matrix and ROC curve on training dataset (B) confusion matrix and ROC curve on testing dataset. Additionally, the dashed line in the ROC curve represents the reference line for random classification (AUC = 0.5).
