# Supplementary material for: Explainable AI-Based Skin Cancer Detection Using CNN, Particle Swarm Optimization and Machine Learning
Source: J Imaging. 2024 Dec 22;10(12):332. doi: 10.3390/jimaging10120332 (PMC11727770; doi:10.3390/jimaging10120332)
Supplement: Supplementary file 1 [file jimaging-10-00332-s001.zip › jimaging-3364029-supplementary.pdf]

Article

Supplementary Material

Explainable AI-Based Skin Cancer Detection Using CNN, Particle Swarm Optimization and Machine Learning

Syed Adil Hussain Shah [1,2,†], Syed Taimoor Hussain Shah [2,*,†], Roa'a Khaled [3], Andrea Buccoliero [1,4], Syed Baqir Hussain Shah [5], Angelo Di Terlizzi [1], Giacomo Di Benedetto [6] and Marco Agostino Deriu [2,*]

1 Department of Research and Development (R&D), GPI SpA, 38123 Trento, Italy; syedadilhussain.shah@gpi.it (S.A.H.S.); andrea.buccoliero@gpi.it (A.B.); angelo.diterlizzi@gpi.it (A.D.T.)
2 PolitoBIOMed Lab, Department of Mechanical and Aerospace Engineering, Politecnico di Torino, 10129 Turin, Italy
3 Department of Computer Engineering, University of Cádiz, 11519 Puerto Real, Spain; roaa.khaled@gm.uca.es
4 Human Science Department, Università degli studi di Verona, Lungadige Porta Vittoria, 17, 37129 Verona, Italy
5 Department of Computer Science, COMSATS University Islamabad (CUI), Wah Campus, Wah 47000, Pakistan; bakirhussain6@gmail.com
6 7HC SRL, 00198 Rome, Italy; giacomo@7hc.tech
* Correspondence: taimoor.shah@polito.it (S.T.H.S.); marco.deriu@polito.it (M.A.D.); Tel.: +39-351-7984023 (S.T.H.S.)
† These authors contributed equally to this work.

S3. Materials and Methods

S3.4. Feature selection using PSO

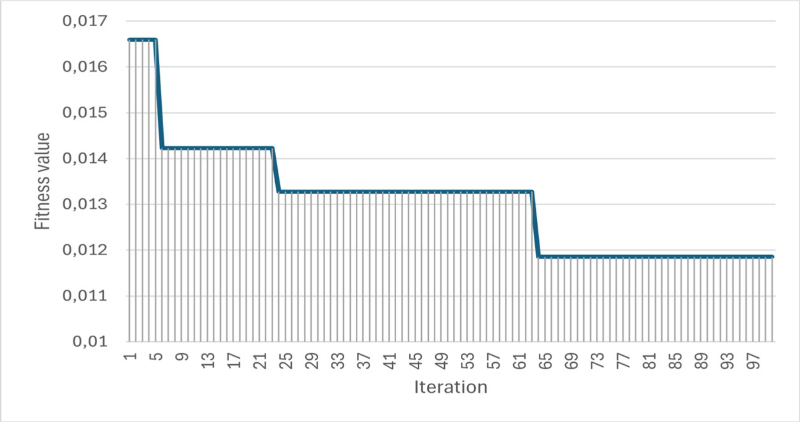

Figure S1 The Evolution of fitness values throughout the PSO learning.

S4. Results

S4.1. Performance evaluation metrics

In the below mathematical equations (Seq 1 to 5), TPV (True Positive Value) term signifies the count of correctly predicted positive instances, while TNV (True Negative Value) indicates the count of accurately predicted negative instances. Conversely, FPV (False Positive Value) denotes the count of negative instances erroneously classified as positive, and FNV (False Negative Value) represents the count of positive instances incorrectly classified as negative.

These metrics serve as fundamental components in assessing the performance of classification models, offering a comprehensive understanding of their predictive accuracy and capability to discern between classes.

$$Acc = \frac{TPV+TNV}{(TPV+FPV)+(TNV+FNV)} \quad \text{(Seq 1)}$$

$$Sen = \frac{TPV}{(TPV+FPV)} \quad \text{(Seq 2)}$$

$$Spe = \frac{TNV}{(FPV+TNV)} \quad \text{(Seq 3)}$$

$$Pre = \frac{TPV}{(TPV+FPV)} \quad \text{(Seq 4)}$$

$$F1 - Score = \frac{2TPV}{(2TPV+FPV+FNV)} \quad \text{(Seq 5)}$$

S4.2. Comparison with Other CNN Models

To identify the most suitable architecture for skin cancer classification, we evaluated several pre-trained CNN models, including Xception, Inception-V3, EfficientNet, and MobileNet, on key metrics such as accuracy, sensitivity, specificity, precision, and F1 score for both validation and testing datasets as mentioned in **Table S*1***. Inception-V3 achieved the highest validation accuracy of 94.6%, slightly outperforming Xception's 94.1%. However, Xception demonstrated superior testing accuracy (89.2%) compared to Inception-V3 (88.3%), EfficientNet (83.9%), and MobileNet (80.4%), indicating its better generalization ability on unseen data.

In addition to performance, we analyzed the computational complexity of each model in terms of the number of layers and trainable parameters. The Xception network consists of 36 convolutional layers and approximately 22 million parameters, making it more efficient compared to Inception-V3, which has 48 layers and approximately 23.9 million parameters. EfficientNet, while achieving reasonable performance (83.9% testing accuracy), has 66 layers and roughly 20 million parameters, making it less efficient due to its more complex architecture. MobileNet, the smallest model in the comparison, contains 28 layers and around 4.2 million parameters, but its reduced size compromises its performance, as reflected in its lower testing accuracy (80.4%) and F1 score (84.4%).

The Xception model strikes an optimal balance between computational complexity and performance, achieving high accuracy while maintaining efficiency. This is particularly advantageous for real-world applications where computational resources are limited, and faster inference is required. Based on these findings, we selected Xception for this experiment, as it provided the best trade-off between performance metrics and computational cost.

Table S1 Performance comparison of various pre-trained CNN models (EfficientNet, Inception-V3, MobileNet, and Xception) for skin cancer classification, including metrics (Acc, Sen, Spe, Pre, F1) for validation and testing datasets, along with the number of layers.

CNN model	Acc (%)	Sen (%)	Spe (%)	Pre (%)	F1 (%)	Number of Convolutional Layers	Number of Parameters (millions)
MobileNet	92.5/80.4	92.5/74.5	92.6/95.2	93.9/97.5	93.2/84.4	66	~20
EfficientNet	93.7/83.9	92.4/79.6	95.5/91.8	96.5/94.7	94.4/86.5	48	~23.9
Xception	94.1/89.2	95.9/93.9	92.1/84.5	93.2/85.8	94.5/89.7	28	~4.2
Inception-V3	94.6/88.3	92.1/85.1	98.1/93.3	98.5/95.2	95.2/89.9	36	~22

S4.3. Ablation Study of the Xception Network

To further refine the Xception network, we conducted an ablation study by freezing different percentages of its 173 total layers: 25%, 50%, 75%, and 100%. The performance metrics for each configuration are detailed in Table S2. Freezing 25% of the layers resulted in an accuracy of 92.0%, with performance gradually improving as more layers were frozen, as displayed in Figure S2. Freezing 50% achieved an accuracy of 93.3%, while freezing 75% and 100% yielded comparable results, with accuracy scores of 94.3% and 94.1%, respectively, as illustrated in Figure S3, Figure S4, and Figure S5.

In terms of sensitivity, specificity, and F1 score, the 75% frozen configuration showed slightly better results (94.8% F1 score) than the fully frozen model (94.5% F1 score). However, the differences were marginal. Given the negligible performance gap and the significant reduction in computational complexity, we opted for the 100%-frozen Xception model for subsequent experiments. Freezing all layers while only adding a few task-specific layers for transfer learning effectively reduced training time and computational resources, making this approach efficient without compromising performance.

This ablation study highlights the importance of optimizing transfer learning strategies to balance performance and computational efficiency. By freezing all layers and focusing on fine-tuning a minimal number of parameters, the Xception network achieves excellent performance with significantly reduced training complexity, making it a robust choice for large-scale experiments.

Table S2 Ablation study results for freezing different percentages of Xception's 173 layers, with metrics (Acc, Sen, Spe, Pre, F1) for validation and testing datasets. The 100% freezing configuration was chosen for its comparable performance and reduced computational cost.

**Freezing layer percentage**	**No. of freezed layers**	**Acc (%)**	**Sen (%)**	**Spe (%)**	**Pre (%)**	**F1 (%)**
25%	42	92.0/84.3	94.5/81.8	89.2/88.3	90.6/91.6	92.5/86.5
50%	85	93.3/88.0	95.2/85.2	91.1/92.3	92.4/94.4	93.8/89.5
75%	127	94.3/89.5	94.6/87.9	93.9/91.7	94.9/93.6	94.8/90.7
100%	173	94.1/89.2	95.9/93.9	92.1/84.5	93.2/85.8	94.5/89.7

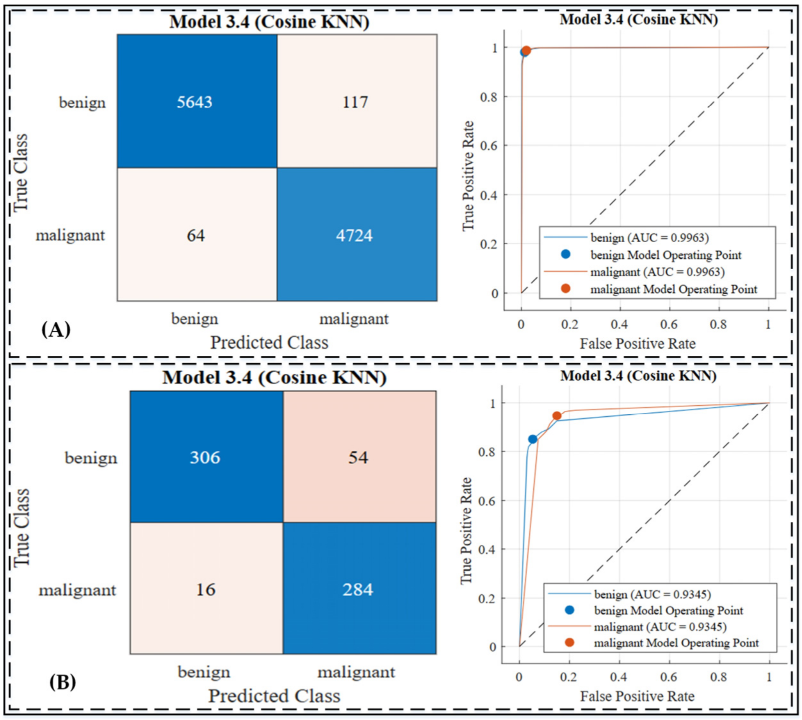

Figure S2 Confusion matrix and ROC curve of experiment 2 by Cosine-KNN classifier: (A) Confusion matrix and ROC curve on training dataset (B) confusion matrix and ROC curve on testing dataset. Additionally, the dashed line in the ROC curve represents the reference line for random classification (AUC = 0.5).

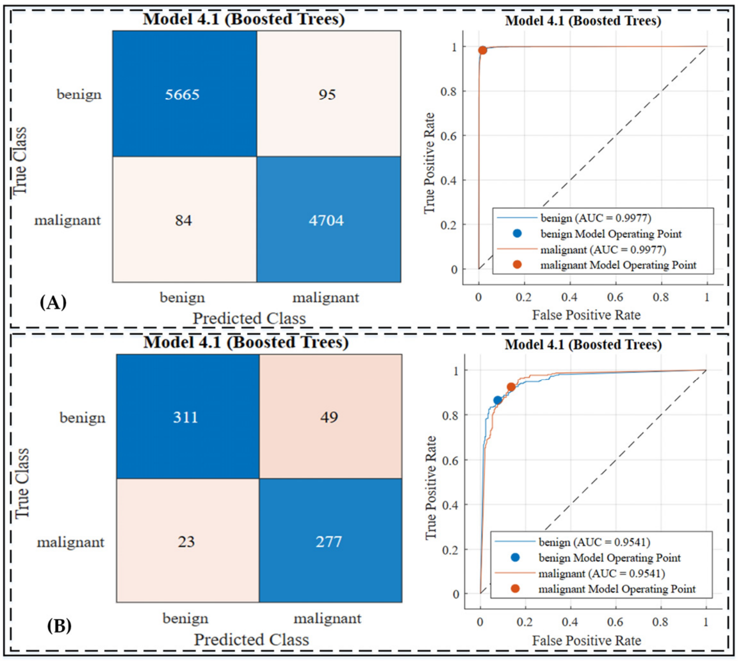

Figure S3 Confusion matrix and ROC curve of experiment 2 by Ensemble-Boosted tree classifier: (A) Confusion matrix and ROC curve on training dataset (B) confusion matrix and ROC curve on testing dataset. Additionally, the dashed line in the ROC curve represents the reference line for random classification (AUC = 0.5).

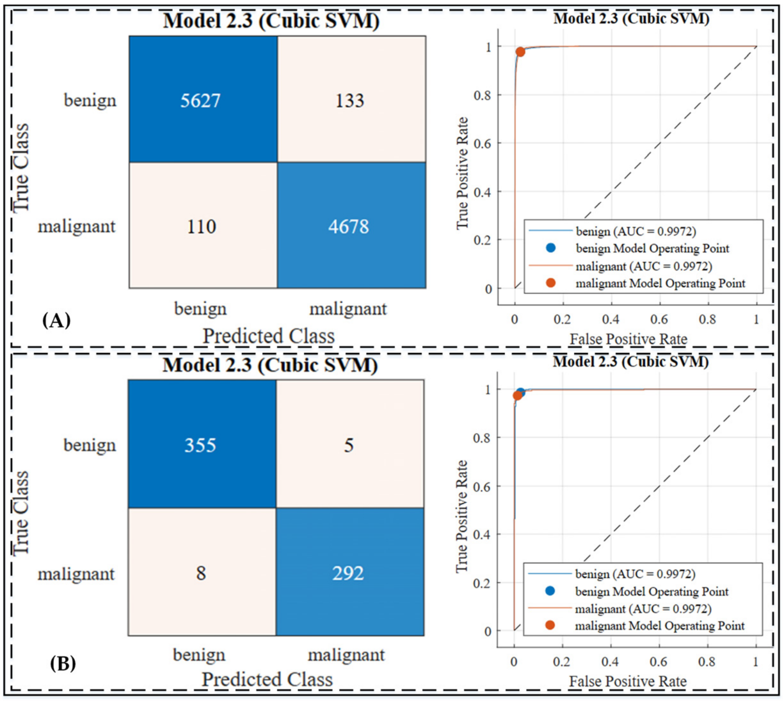

Figure S4 Confusion matrix and ROC curve of experiment 3 by Cubic SVM classifier: (A) Confusion matrix and ROC curve on training dataset (B) confusion matrix and ROC curve on testing dataset. Additionally, the dashed line in the ROC curve represents the reference line for random classification (AUC = 0.5).

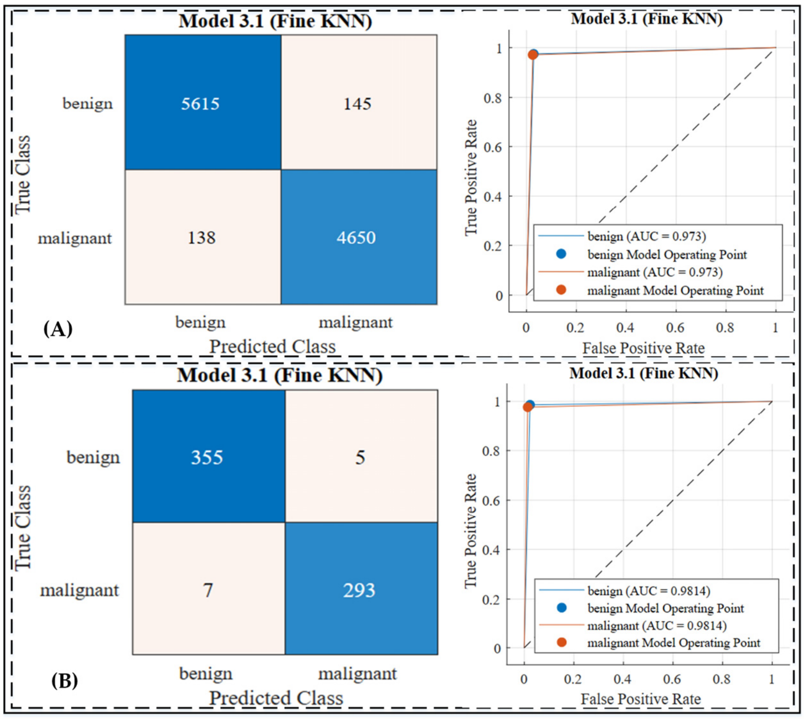

Figure S5 Confusion matrix and ROC curve of experiment 3 by Fine-KNN classifier: (A) Confusion matrix and ROC curve on training dataset (B) confusion matrix and ROC curve on testing dataset. Additionally, the dashed line in the ROC curve represents the reference line for random classification (AUC = 0.5).

Nomenclature

**Term/Abbreviation**	**Definition**
**AI**	Artificial Intelligence
**XAI**	Explainable Artificial Intelligence
**CNN**	Convolutional Neural Network
**Xception**	A CNN architecture based on depthwise separable convolutions
**EfficientNet**	A family of CNN architectures optimized for performance and efficiency
**MobileNet**	A lightweight CNN architecture designed for mobile and embedded applications
**Inception-V3**	A CNN architecture known for its efficient multi-scale feature extraction
**ISIC 2018 Dataset**	A publicly available dataset for skin lesion analysis and classification tasks
**HAM10000 Dataset**	A dataset comprising 10,015 dermoscopic images for skin cancer diagnosis
**PSO**	Particle Swarm Optimization, an optimization algorithm inspired by the social behavior of particles
**Grad-CAM**	Gradient-Weighted Class Activation Mapping, a technique for visualizing model attention
**LIME**	Local Interpretable Model-Agnostic Explanations, a technique for generating interpretable explanations
**Occlusion Sensitivity**	A technique to measure the impact of occluding specific parts of an image on model predictions
**Softmax**	An activation function used in the final layer of neural networks for multi-class classification
**Subspace KNN**	An ensemble method combining k-Nearest Neighbors classifiers on feature subspaces
**Ablation Study**	A method for evaluating the impact of modifying or removing components of a model
**Accuracy (Acc)**	The ratio of correctly classified instances to the total number of instances
**Sensitivity (Sen)**	The ability of a model to correctly identify positive (malignant) cases
**Specificity (Spe)**	The ability of a model to correctly identify negative (benign) cases
**Precision (Pre)**	The proportion of true positives among all predicted positives
**F1 Score (F1)**	The harmonic mean of precision and sensitivity
**ROC Curve**	Receiver Operating Characteristic curve, a plot illustrating the diagnostic ability of a classifier
**AUC**	Area Under the Curve, a metric derived from the ROC curve indicating model performance
**SVM**	Support Vector Machine, a supervised machine learning algorithm
**Linear SVM (L-SVM)**	An SVM classifier with a linear kernel
**Quadratic SVM (Q-SVM)**	An SVM classifier with a quadratic kernel
**Cubic SVM (C-SVM)**	An SVM classifier with a cubic kernel
**Fine Gaussian SVM (FG-SVM)**	An SVM classifier with a fine Gaussian kernel
**Medium Gaussian SVM (MG-SVM)**	An SVM classifier with a medium Gaussian kernel

**Coarse Gaussian SVM (CG-SVM)**	An SVM classifier with a coarse Gaussian kernel
**Fine KNN (F-KNN)**	A k-Nearest Neighbors classifier with a small neighborhood size
**Medium KNN (M-KNN)**	A k-Nearest Neighbors classifier with a medium neighborhood size
**Coarse KNN (C-KNN)**	A k-Nearest Neighbors classifier with a large neighborhood size
**Cosine KNN (Cos-KNN)**	A k-Nearest Neighbors classifier using cosine distance as the metric
**Weighted KNN (W-KNN)**	A k-Nearest Neighbors classifier that assigns weights to neighbors
**Boosted Trees Ensemble (BT-Ensemble)**	An ensemble learning method that combines weak learners into a strong classifier
**Bagged Trees Ensemble (BagT-Ensemble)**	An ensemble method using bootstrap aggregation to improve classifier performance
**Subspace Discriminant Ensemble (SD-Ensemble)**	An ensemble classifier that trains discriminant subspace models
**Subspace KNN Ensemble (SK-Ensemble)**	An ensemble method combining multiple KNN models across subspaces
**RUSBoosted Trees Ensemble (RBT-Ensemble)**	A variation of Boosted Trees designed for imbalanced datasets using Random Under Sampling
**Feature Extraction**	The process of deriving meaningful representations from raw data
**Feature Selection**	The process of selecting the most relevant features from a dataset
**Dimensionality Reduction**	Techniques to reduce the number of features while retaining significant information
**5-Fold Cross-Validation**	A method to evaluate model performance by splitting data into five subsets, using four for training and one for testing in each iteration
**Benign**	Non-cancerous skin lesions, includes bkl, df, nv, and vasc classes.
**Malignant**	Cancerous skin lesions, includes akiec, bcc, and mel classes.
**Seq**	Supplementary file-based equation
**Figure S**	Figure is provided in Supplementary file.
**akiec**	Actinic keratoses and intraepithelial carcinoma.
**bcc**	Basal cell carcinoma.
**bkl**	Benign keratosis-like lesions.
**df**	Dermatofibroma.
**mel**	Melanoma.
**nv**	Melanocytic nevi.
**vasc**	Vascular lesions.
